# Peer Review of “A Local Community-Based Social Network for Mental Health and Well-being (Quokka): Exploratory Feasibility Study”

**DOI:** 10.2196/33925

**Published:** 2021-10-27

**Authors:** Maria da Graça Campos Pimentel

**Affiliations:** 1 Departamento de Ciências de Computação Universidade de São Paulo São Carlos, SP Brazil

**Keywords:** local social network, community health, well-being, digital health, consumer health

*This is a peer-review report submitted for the paper “A Local Community-Based Social Network for Mental Health and Well-being (Quokka): Exploratory Feasibility Study.”*

## Round 1 Review

### General Comments

The authors present a study [1] evaluating the impact of offering college students challenges designed to develop healthy habits. The challenges were offered to students in 4 campuses via a community-based social network. The challenges follow the same structure: volunteers in each campus customize them for their community. The “community-based” research involved partnerships with local mental health resources and services available, local businesses, or school-affiliated groups. The study aimed at evaluating the preferences of the participants relative to three features: (a) local versus remote activities, (b) group versus individual activities, (c) new versus familiar activities.

### Specific Comments

#### Major Comments

1. Do the challenges correspond to the “interventional program that makes use of the established success of community-based social programs for behavior change”? Or the “community-based social network”?

2. Given the variety and duration of the challenges and the intervention’s length, were the participants expected to accumulate the “change in habits” over the overall period? And after that?

3. How were the campuses selected?

4. How much of the study could be generalized to different campuses? Please present the “typical” demographics in these campuses (students, staff, and locals) and discuss their impact on the study.

5. How were the volunteers in each campus selected?

6. Are there any data on the demographics of the participants?

7. Please review the text for clarity and precision. In particular:

(a) The hypotheses H1-H3 (presented in the section *Study Design*) do not match the first result reported in the section *Evaluation Outcomes*.

(b) The hypothesis rejected in the study is never presented as a hypothesis (you may want to refer to H1).

(c) Several portions of the text are repetitive.

#### Minor Comments

8. Please revise the style to substitute imprecise terms.

9. Please reconsider employing section headers for the challenges in the section *Challenges Themes*.

## Round 2 Review

### General Comments

The authors provide answers to reviewers’ questions and present a revised and improved version that tackles their comments. I have a few concerns that I suggest the authors tackle in a new revision, as detailed below.

### Specific Comments

#### Major Comments

1. The authors included a related work section after the Introduction. Following the IMRD structure suggested in JMIR’s Instructions for Authors document, I suggest that the motivating literature be presented, instead, in the *Introduction* itself.

2. The authors do not compare their contribution with prior work, limiting the potential impact of their approach. I suggest including a *Comparison With Prior Work* section in the *Discussion* section, as suggested in the JMIR's Instructions for Authors document.

3. When comparing your contribution with that of prior work, please specify how you identified the prior work selected for discussion. Please make sure to include recent work.

4. How did you select the work currently discussed in the “Related Section”?

#### Minor Comments

5. I suggest modifying the title to make it explicit that “well-being” is related to (mental) health.

## Round 3 Review

### General Comments

The authors responded to reviewers’ questions and acted on their suggestions. However, the resulting manuscript still has issues regarding its structure concerning the problems tackled and the work carried out. My main concerns follow.

### Specific Comments

#### Major Comments

1. You propose a system to tackle a problem and then evaluate the system. In my humble opinion, the system itself pertains to the “method” you used to assess whether or not you contributed to the problem. Is this the case? If so, please revise the manuscript structure accordingly.

2. The revised version is annotated, which makes it very hard to follow the resulting manuscript. Accepting all changes exposes (a) contents not aimed at the reviewers, and (b) formatting issues.

3. Please avoid using sections with a single paragraph (as in challenging themes): the result is distracting.
